# Proceedings or the Almakee Co. Medical Society

**Published:** 1873-07-01

**Authors:** S. H. Drake

**Affiliations:** Secretary; Waukon, Mich.


### PROCEEDINGS OF THE ALLAMAKEE COUNTY MEDICAL SOCIETY.

WAUKON, Mich., May 8th, 1873.

Pursuant to the adjournment of a previous meeting, a number of the physicians of Allamakee county met at Waukon, May 7, 1873, for the purpose of forming a permanent Medical Association for the county, Dr. H. E. McNult, of Dorchester, in the chair.

A Constitution and By-Laws were presented, approved and signed by the gentlemen present, viz.:

J. S. Green, Luther Brown, Postville; H. E. McNult, Dorchester; S. H. Drake, Rossville; W. C. Earle, T. H. Barns, Waukon; B. E. Brockhansen, E. Stuger, Lansing.

On ballot the following members were elected officers of the Society:

T. H. Barnes, President; E. Stuger, Vice-President; S. H. Drake, Secretary; W. C. Earle, Treasurer; J. L. Green, H. E. McNult, B. E. Brockhansen, Board of Censors.

On motion, Dr. Hedge, of Waukon, was unanimously elected an honorary member of the Society.

The Secretary was authorized to furnish credentials to any member desiring to attend the State Medical Society. The Fee-bill of the North Iowa Medical Society was read by the Secretary and adopted, with the exception of mileage, which was fixed at fifty cents per mile in the day time, the rate at night remaining unchanged.

Drs. Brown, McNult and Brockhansen were appointed Essayists by the President.

The following subject was presented by Dr. Stuger for discussion at the next meeting: "The Physiological Effects and Therapeutical Value of Alcohol."

On motion, it was decided to hold the annual meetings at Waukon on the first Wednesday of May of each year, the place of holding the other meetings to be decided by vote of the Society.

On motion, the members of the Society were authorized to strike a balance of their accounts at the end of every quarter, viz. : At the end of March, June, September and December, and all balance due at such times shall draw one-half per cent. per annum.

On motion, the Secretary was directed to have the proceedings of the Society published in the Postville *Review*, Lansing *Journal*, Waukon *Standard*, and Chicago MEDICAL EXAMINER.

Adjourned to meet in Waukon on the first Wednesday of September, 1873.

S. H. DRAKE, *Secretary*.

